# Soil Solarization Efficiently Reduces Fungal Soilborne Pathogen Populations, Promotes Lettuce Plant Growth, and Affects the Soil Bacterial Community

**DOI:** 10.3390/biology13080624

**Published:** 2024-08-15

**Authors:** George T. Tziros, Anastasios Samaras, George S. Karaoglanidis

**Affiliations:** Laboratory of Plant Pathology, Forestry and Natural Environment, Faculty of Agriculture, Aristotle University of Thessaloniki, P.O. Box 269, 54124 Thessaloniki, Greece; gtziros@yahoo.gr (G.T.T.); samarasanast@gmail.com (A.S.)

**Keywords:** leafy vegetables, next-generation sequencing, NGS, soil solarization, soilborne disease management

## Abstract

**Simple Summary:**

The impact of various soil disinfestation methods applied to soil was evaluated in a commercial field that has been repeatedly used for lettuce cultivation for two successive years. The populations of four soilborne pathogens, evaluated via RT-qPCR before and after the implementation of the specific methods, showed that all the evaluated methods significantly reduced the populations of the specific soilborne pathogens. More specifically, soil solarization was observed to be the most effective method. Amplicon sequencing analysis of 16S rRNA-encoding genes showed that the soil bacterial microbiomes were influenced by the implementation of the various soil treatments.

**Abstract:**

Lettuce is the most cultivated leafy vegetable in Greece; however, due to the adopted intensive cropping system, its cultivation is susceptible to many soilborne pathogens that cause significant yield and quality losses. In the current study, the impact of various soil disinfestation methods such as solarization, chemical disinfestation, and application of a biofungicide were evaluated in a commercial field that has been repeatedly used for lettuce cultivation. The populations of soilborne pathogens *Rhizoctonia solani*, *Pythium ultimum*, *Fusarium oxysporum,* and *Fusarium equiseti* were measured via qPCR before and after the implementation of the specific disinfestation methods. Although all the tested methods significantly reduced the population of the four soilborne pathogens, soil solarization was the most effective one. In addition, solarization reduced the number of lettuce plants affected by the pathogens *R. solani* and *F. equiseti*, and at the same time, significantly influenced the growth of lettuce plants. Amplicon sequence analysis of 16S rRNA-encoding genes used to study the soil bacterial community structure showed that Firmicutes, Proteobacteria, and Actinobacteria were the predominant bacterial phyla in soil samples. In general, solarization had positive effects on Firmicutes and negative effects on Proteobacteria and Actinobacteria; soil fumigation with dazomet increased the relative abundance of Firmicutes and Proteobacteria and reduced the corresponding values of Actinobacteria; and biofungicide had no significant effects on the three predominant bacterial phyla. The bacterial community composition and structure varied after the application of the soil disinfestation treatments since they imposed changes in the α- and β-diversity levels. The results of this study are expected to contribute towards implementing the most effective control method against the most common soilborne pathogens in intensively cultivated fields, such as those cultivated with leafy vegetables.

## 1. Introduction

Lettuce is one of the most consumed leafy vegetables in Greece, primarily field-grown or under protection in multi-tunnels or in greenhouses enabling year-round cultivation [1]. However, as leafy vegetables are cultivated intensively, mostly without crop rotation in successive cycles every year in the same field, they are susceptible to several plant pathogens causing foliar and soilborne diseases [2].

In Greece, damping-off symptoms on lettuce grown as baby leaves have been associated with *Rhizoctonia solani* and three *Pythium* spp. (*P. ultimum*, *P. aphanidermatum,* and *P. sylvaticum*) [1]. Lettuce Fusarium wilt caused by *Fusarium oxysporum* f. sp. *lactucae* [3] and an emerging foliar disease on the same crop caused by *Fusarium equiseti* [4] have also been reported recently in the country. Such soilborne pathogens survive via over-seasoning structures in the soil or on crop debris and cause severe yield losses by infecting the plants at several growth stages. 

Soil disinfestation based on non-selective chemical or physical means is used as the main method to eradicate the initial inoculum of soilborne pathogens, preventing infection at the early stages of cultivation and disease development [5]. However, nowadays, such practices should have fewer effects on the physical and chemical properties of the soil and at the same time maintain the biological balance [5]. Furthermore, soil fumigants should be applied a sufficiently long time before planting to avoid phytotoxic effects on the cultivated plants [6]. On the other hand, solarization has rarely been associated with negative side effects—such as phytotoxicity and pathogen reinfestation—because of the creation of a biological vacuum, and is usually accompanied by induced suppressiveness in which re-establishment of the pathogen is prevented after the application of the specific practice [7]. 

Solarization is applied during summer and can increase the temperature in the upper soil layers to sub-lethal or even lethal levels for soilborne pathogens [5]. However, bacteria such as those of the genus Bacillus are considered less sensitive to high temperatures than fungi, and thus survive solarization and could have a crucial role in disease suppressiveness in solarized soils [8]. The combination of solarization with soil fumigants leads to enhanced disease control, compared to each practice alone, since the volatiles are captured under the plastic film and thus could distribute in the soil for a longer period. Such combination can shorten the period that is necessary for solarization and reduce the application rate of the fumigants [5]. In addition, the application of biocontrol agents against soilborne pathogens is considered to be effective after chemical disinfestation or solarization as they prevent the so-called biological vacuum in the soil, contribute to its biological activity, and eventually increase plant production [9].

The microbial structure and diversity, which are associated with the soil ecosystem function [10], are used as indicators to examine the integrity of the soil microbiome [11]. However, chemical soil fumigants and their degradation metabolites could have negative effects on beneficial soil microbial communities [12,13]. Such microorganisms, prevalent in healthy soils, produce a variety of antimicrobial compounds to suppress soilborne pathogens [14,15] and limit pathogen invasion through niche competition [16,17]. In addition, soil disinfestation induces microbial changes in the soil—especially in the rhizosphere and plant roots—and affects the chemical, physical, and biological properties of soil, and consequently, plant growth is also affected [18].

Several traditional and emerging fungal diseases cause significant yield losses on leafy vegetables grown for fresh or else ready-to-eat salads. Considering that a limited number of chemicals are registered for use in these crops as well as the impact of soil fumigation on the environment, the management of soilborne pathogens based on alternative methods is a real challenge. Hence, this study was initiated aiming to assess the impact of different soil disinfestation methods (chemical, physical, and biological) on the following: (a) the fungal population density of four major soilborne pathogens of lettuce (*R. solani*, *P. ultimum*, *F. oxysporum*, and *F. equiseti*); (b) the disease incidence caused by *R. solani* and *F. equiseti*; (c) the growth of lettuce plants; and (d) the bacterial microbiome responses of the treated soil using NGS analysis.

## 2. Materials and Methods

### 2.1. Field Study Experimental Set Up

The field experiment was established at a commercial farm specialized in the cultivation of leafy vegetables (Vezyroglou Farm, Alexandria, Imathia, Central Macedonia, Greece) for two consecutive years (2019 and 2020). During both years, four different treatments were evaluated, with two replications each in a completely randomized block design. The experimental plots were 10 m × 36 m (2019) or 5 m × 17 m (2020) in size.

In 2019, the tested treatments were the following: (1) soil solarization carried out by laying a three-layered 30 to 35-micrometer-thick transparent polyethylene film (Orgasun, Plastika Kritis, Greece) on the soil for 50 days; (2) the granular fumigant dazomet (Basamid 98 GR, K&N Efthymiadis, Greece) applied at a dose rate of 60 g active ingredient per m^2^ and incorporated at a depth of 30 cm through disking. After the application, treated plots were irrigated and the soil surface was covered with the same plastic film applied in the solarization treatment for 50 days; (3) the commercial *Coniothyrium terricola*-based biofungicide product (hereafter CF), which also contained in lower concentrations the beneficial bacteria *Bacillus psychrodurans* and *B. licheniformis* (Re-Genera 2.0 SL, Geenea, Italy) in a total concentration of 1 × 10^9^ cfu/mL, applied by mixing 0.5 mL per m^2^ into the top soil twice (the first application was conducted at the time of soil solarization initiation and the second at the time of removal of the plastic film); and, (4) the untreated control. In the second year of the experiment (2020), the tested treatments were as follows: (1) soil solarization applied as previously described; (2) commercial biofungicide product Re-Genera applied as previously described; (3) the combined application of solarization for 50 days and the biofungicide Re-Genera applied once 5 days after the removal of the plastic film; and (4) the untreated control.

After completing all the experimental applications, the soil was plowed to a depth of approximately 20 cm. Lettuce plants of Romaine type (cv. Maximus) at the stage of 4–6 true leaves (BBCH 104–106) were transplanted in the field the following day and cultivated under common agricultural practices, without receiving any fungicide application. In each experimental plot there were five planting beds, 1.8 m wide and 36 m (2019) or 17 m (2020) long, separated by a 0.2-meter-long passageway, and each one consisting of 4 planting rows, making a total of 20 planting rows per experimental plot.

### 2.2. Soil Sampling and DNA Extraction from the Soil

Soil samples were collected from each experimental plot twice; one day before the application of each individual treatment and one day before the transplanting of lettuce plants after the end of the treatments. Five soil samples were collected from each raised bed of each experimental plot, at 6 m from the other, using a soil auger. Each sample was obtained at a depth of 0 to 40 cm and weighed approximately 500 g. In total, 25 individual soil samples were collected from each experimental plot and combined into one pooled sample. Soil samples were not obtained from the two external lanes of each experimental plot to minimize possible influence from adjacent treatments. Soil samples were thereafter transported to the laboratory in individual polyethylene bags and directly subjected to the DNA extraction process.

DNA was extracted with the DNeasy Power Soil kit (Qiagen, Hilden, Germany) following the manufacturer’s instructions and its concentration was measured using a P330 nanophotometer (Implen GmbH, Munich, Germany). All DNA extracts were stored at −20 °C until further processing.

### 2.3. Quantification of Soilborne Pathogens with qPCR

The effect of the tested treatments on the populations of the four most common soilborne pathogens (*R. solani*, *P. ultimum*, *F. oxysporum,* and *F. equiseti*) was determined using real-time quantitative polymerase chain reaction (RT-qPCR) assays. To quantify the *R. solani* inoculum, the ITS1-5.8S-ITS2 region was amplified with primers ST-RS1 and ITS4 [19,20]. Amplification conditions consisted of a single cycle at 94 °C for 3 min, followed by 40 cycles of 94 °C for 30 s, 60 °C for 30 s, and 72 °C for 20 s, while the melt curve stage consisted of 94 °C for 1 min, 60 °C for 30 s, and 94 °C for 30 s. *P. ultimum* inoculum was quantified by amplification of the internal transcribed spacer regions (ITS1 and ITS2) using primers PulF2 and PulR2 [21]. Amplification conditions consisted of a single cycle at 95 °C for 3 min, followed by 40 cycles of 95 °C for 15 s, 60 °C for 30 s, and 72 °C for 20 s, while the melt curve stage consisted of 95 °C for 1 min, 55 °C for 30 s, and 95 °C for 30 s. For the quantification of *F. oxysporum* in soil samples, the FOW1 gene was amplified with primers FOW1F and FOW4R [22]. Amplification conditions consisted of a single cycle at 95 °C for 3 min, followed by 40 cycles of 95 °C for 30 s, 52 °C for 30 s, and 72 °C for 20 s. Following the final amplification cycle, a melting curve was acquired, consisting of 95 °C for 1 min, 60 °C for 30 s, and 95 °C for 30 s. The quantification of *F. equiseti* was carried out using a recently developed qPCR protocol including a set of two primers (FeqELf-F and FeqELf-R) and a probe (FeqELf-Pro) [4]. Reaction conditions were as those described in Tziros et al. [4].

The reaction mixture for each DNA sample consisted of 3 μL of DNA template, 10 μL of Universal qPCR Master Mix (New England Biolabs, Ipswich, MA, USA), 500 nM of each of the forward and reverse primers, 1.25 μL of EvaGreen dye (Biotium Inc., Fremont, CA, USA), and 0.1 μL BSA (New England Biolabs, Ipswich, MA, USA) in a final volume of 20 μL. For each sample, triplicate reactions were performed and molecular-grade water (Panreac, AppliChem, Barcelona, Spain) was used as non-template control. The reactions were performed using a Strategene Mx3005P qPCR System (Agilent Technologies, Santa Clara, CA, USA) on 96-well plates. Data were collected in the last holding stage of each cycle and the results were analyzed using the Strategene MxPro-Mx3005P Software (Version 4.00; Agilent Technologies).

The amount of each pathogen (number of copies) in 1 g of dry soil was calculated by incorporating the mean Ct value into the standard curve equation. Standard curves were generated for the quantification of *R. solani*, *P. ultimum,* and *F. oxysporum* following the procedure described recently by the authors for the preparation of the standard curve of *F. equiseti* [4].

### 2.4. Disease Assessment

The damping-off incidence caused by *R. solani* was assessed by recording the total number of plants exhibiting visual damping-off symptoms and/or the number of dead plants [23] throughout the individual experimental plots at the early stages of development of lettuce plants, 7 and 21 days after planting. Damping-off symptoms were associated to *R. solani* by transferring the symptomatic plants to the lab and subsequently isolating the pathogen following the procedure described by Tziros and Karaoglanidis [1]. 

The assessment of *F. equiseti* incidence was conducted at harvest. A total of 18 lettuce plants were randomly selected from each experimental plot and evaluated for foliar disease symptoms caused by the pathogen by counting the number of leaves exhibiting leaf spot symptoms on each individual plant, as described previously in Tziros et al. [4].

### 2.5. Plant Fresh Weight

At harvest, the mean fresh weight (g) of the plants grown in each experimental plot was measured. In total, 20 randomly selected plants per plot were used for the measurements.

### 2.6. Bacterial Microbiome Analysis

Total genomic DNA extracted from soil samples, collected before and after the application of the different evaluated soil disinfestation treatments during the first growing season (2019), were used to amplify the V4 region of the 16S rRNA gene of soil bacteria. The concentration of the extracted DNA was measured using a P330 nanophotometer (Implen GmbH) and the quality was determined on 0.8% agarose gel. Three DNA extracts from each experimental plot were shipped to BGI Tech Solutions Co., Ltd. (Hong Kong) for 16S-V4 Amplicon Sequencing with Package HiSeq2500. Amplification, library preparation, and sequencing were performed by BGI Co., Ltd.

### 2.7. Bioinformatic Analysis

Raw sequencing reads were first quality-checked using FastQC (0.12.0) to assess the quality of the generated sequences [24]. Adapter sequences and low-quality bases were trimmed from the raw reads using Trimmomatic (v0.39), retaining sequences longer than 150 bp. The raw data were filtered to eliminate the adapter pollution and low quality to obtain clean reads; then, paired-end reads with overlap were merged to tags [25]. These tags were clustered to operational taxonomic units (OTUs), defined as groups that showed sequence similarities greater than 97% [26]. Taxonomic ranks were assigned to the (OTUs) representative sequence using Ribosomal Database Project (RDP) Naïve Bayesian Classifier v.2.2 [27]. Alpha diversity (α-diversity), beta diversity (β-diversity), and the different species’ screening were analyzed based on OTU and taxonomic ranks [28]. The following α-diversity indices were calculated to assess within-sample diversity, utilizing the microbiome package Mothur (v1.31.2; University of Michigan, Ann Arbor, MI, USA): observed species richness, Chao1, the abundance-based coverage (ACE) species richness estimators, the Shannon diversity index, and the Inverse Simpson diversity index [29]. The corresponding rarefaction curve for the Shannon diversity index was drawn using the software package R (v3.1.1). The shifts in bacterial community composition were visualized using principal component analysis (PCA) of the OTUs using the relative abundance values.

For beta-diversity analysis, Bray–Curtis dissimilarity and UniFrac distances were computed, and Principal Coordinate Analysis (PCoA) was performed to visualize the differences among the various applied treatments [30]. The commonly used Bray–Curtis distance value index was used to reflect the differences between two communities, ranging between values from zero to one (a zero Bray–Curtis value represents an exactly similar community structure) [31]. Heat map analysis was performed based on the relative abundance of OTUs in each treatment (before and after their application). To minimize the degree of difference in the relative abundance value, the values were all log-transformed, except for the class level in which the absolute unlogged relative abundance values were used. Differential abundance analysis between sample groups was conducted using the DESeq2 package. Heat maps were generated with R v4.1.2 using the R packages heatmap v1.0.12 and ggplot2 v3.3.3 [32]. The default parameters of the heatmap were used to construct the dendrograms. Dendrograms were generated using the Euclidean distance measure and the complete clustering method. All statistical analyses were performed using R (v3.1.1), with significance set at *p* < 0.05 [33].

The sequencing data were submitted to the Sequence Read Archive of NCBI with bioproject accession number PRJNA1022862.

### 2.8. Statistical Analysis

qPCR data for each soilborne pathogen, plant fresh weight, disease incidence values, and α-diversity indices were subject to a one-way analysis of variance (ANOVA) performed using SPSS v25.0 (SPSS Inc., Chicago, IL, USA). Mean values of the differences before and after the implementation of the various treatments were determined using Tukey’s multiple range test at the *p* < 0.05 level.

## 3. Results

### 3.1. Standard Curves 

Standard curves were generated by serial ten-fold dilutions (10^1^ to 10^9^ copies/μL) of in vitro transcripts for each one of the four evaluated pathogens (Figure S1). The standard curve for *R. solani* showed a linear dynamic range of amplification over nine orders of magnitude with a slope of −3.342, corresponding to an efficiency of 99.1%, while the linearity of the curve, denoted by the *R^*2*^* value, was 0.99. The standard curve used for the calculation of the copy numbers of the target gene of *P. ultimum* showed an *R*^2^ value linearity of 0.98, amplification efficiency of 98.3%, with a slope of −3.361. PCR amplification efficiency for the calculation of the copy numbers of the FOW1 gene in the case of *F. oxysporum* was 94.8% with a 0.99 *R*^2^ value and slope of −3.453. The previously generated standard curve for *F. equiseti* had an efficiency of 95.5% with a slope of −3.315, while the linearity of the curve, denoted by the *R^*2*^* value, was 0.99 [4].

### 3.2. Impact of Disinfestation Treatments on Soilborne Pathogen Populations

**Figure 1:**
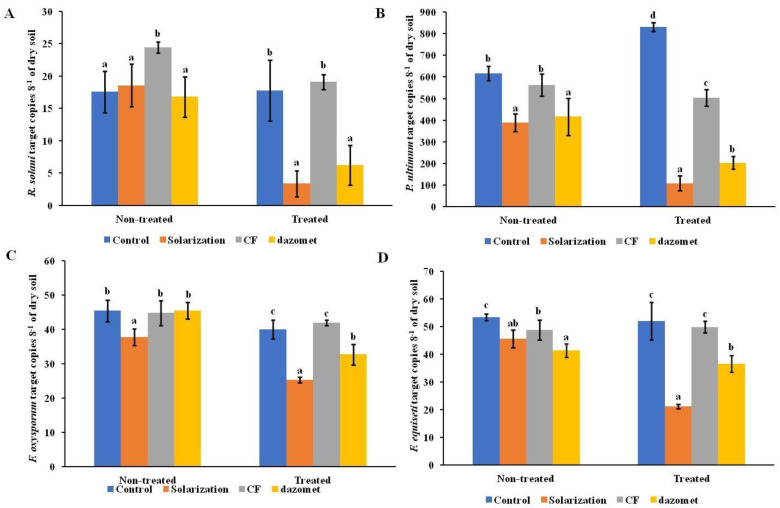
Impact of soil treatments applied in the first year of the experiment (2019) on the population of (**A**) *Rhizoctonia solani*, (**B**) *Pythium ultimum*, (**C**) *Fusarium oxysporum*, and (**D**) *Fusarium equiseti* as determined by quantitative PCR and represented as the number of copies of the corresponding target gene for each of the evaluated soilborne pathogens. Each value is the mean of four biological replicates and two technical replicates for each biological replicate. Error bars represent the standard deviation. Means followed by different letters indicate statistically significant differences within each sampling time; before and after the application of the evaluated treatments (samples collected from non-treated and treated plots, respectively) as defined by Tukey’s test (*p* < 0.05). Non-treated and treated marks indicate sampling time. Control: no treatment; CF: *Coniothyrium*-based biofungicide.

**Figure 2:**
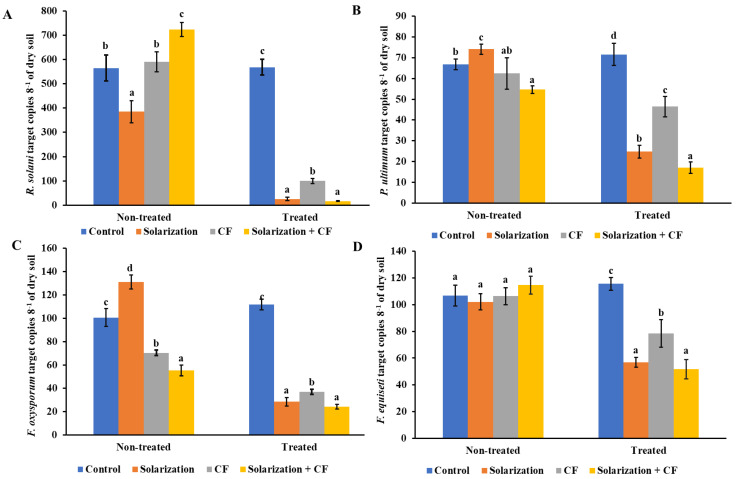
Impact of soil treatments applied in the second year of the experiment (2020) on the population of (**A**) *Rhizoctonia solani*, (**B**) *Pythium ultimum*, (**C**) *Fusarium oxysporum*, and (**D**) *Fusarium equiseti* as determined by quantitative PCR and represented as the number of copies of the corresponding target gene for each of the evaluated soilborne pathogens. Each value is the mean of four biological replicates and two technical replicates for each biological replicate. Error bars represent the standard deviation. Means followed by different letters indicate statistically significant differences within each sampling time; before and after the application of the evaluated treatments (samples collected from non-treated and treated plots, respectively) as defined by Tukey’s test (*p* < 0.05). Non-treated and treated marks indicate sampling time. Control: no treatment; CF: *Coniothyrium*-based biofungicide.

The results of the qPCR showed a significant impact of the treatments applied during both years of the experiment on the population dynamics of *R. solani* (Figure 1 and Figure 2). The application of solarization significantly reduced (*p* < 0.05) the copy numbers of the *R. solani*’ ITS region in soil. In 2019, solarization and the application of dazomet were the most effective treatments in reducing *R. solani* inoculum (Figure 1A). The application of the biological control agent showed no significant reduction in the copy numbers of *R. solani* in comparison to the control treatment (non-treated plots). In the second year of the experiment, solarization significantly reduced the population of *R. solani* when used alone or in combination with the biological control agent compared to the non-treated plots (Figure 2A). In addition, plots treated with the biological control agent showed significantly reduced population dynamics of the specific pathogen in comparison to the untreated control ones, although yielding significantly lower copy numbers when compared to the solarized ones (Figure 2A).

The application of solarization was the most effective treatment (*p* < 0.05) in regards to *P. ultimum* population reduction in the first year of the experiment compared to the non-treated plots, as well as to the plots treated with the chemical fumigant (dazomet) and the biological control agent (Figure 1B). However, a reduction in the *P. ultimum* population density was also observed in plots treated with either dazomet or biofungicide. In 2020, a higher reduction (*p* < 0.05) in the population of *P. ultimum* was observed in plots that received the combined application of solarization and biofungicide (Figure 2B).

The qPCR assays yielded similar results when estimating the impact of the various treatments on the soil population of *F. oxysporum* and *F. equiseti* (Figure 1C,D and Figure 2C,D). The application of solarization showed the most significant reduction in the copy numbers of the *FOW1* and *TEF1-α* genes of *F. oxysporum* and *F. equiseti*, respectively, in the two years of the experiment. In 2019, the application of dazomet reduced the population density of both pathogens, although in lower levels compared to the solarized plots, while the application of the biological control agent showed no significant differences in comparison to the non-treated plots (Figure 1C,D). In the second year of the experiment, the solarization induced a higher reduction (*p* < 0.05) in the population of both *Fusarium* spp. (Figure 2C,D) when applied alone or in combination with the biological control agent. Furthermore, the application of the biological control agent significantly reduced the population of both pathogens in comparison to the control, however, in lower levels compared to the solarized blocks (Figure 2C,D).

### 3.3. Disease Assessment

**Figure 3:**
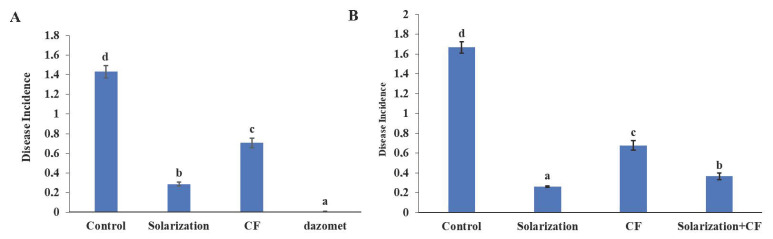
Damping-off—caused by *Rhizoctonia solani*—incidence (%) on lettuce plants grown in plots treated with several soil disinfestation methods during 2019 (**A**) and 2020 (**B**). Error bars represent the standard deviation. Means followed by different letters indicate statistically significant differences among the evaluated treatments as defined by Tukey’s test (*p* < 0.05). Control: no treatment; CF: *Coniothyrium*-based biofungicide.

During both years of the study, only *R. solani* damping-off symptoms and *F. equiseti* leaf symptoms appeared on lettuce plants. The mean percentage of plants exhibiting damping-off symptoms associated with *R. solani* was significantly lower in all treatments compared to the control during both years of the study (Figure 3A,B). In 2019, the application of solarization significantly reduced the disease incidence compared to the control treatment. During the same year, lower disease incidence (*p* < 0.05) was observed in plots treated with dazomet, followed by the solarization treatment (Figure 3A). During 2020, and in the absence of dazomet treatment, solarization was the most effective treatment against *R. solani*, followed by its combination with CF (Figure 3B). CF, when applied alone, significantly reduced the disease incidence as it was 14% less than the control; however, in this treatment, disease incidence was higher (*p* < 0.05) compared to treatments in which solarization was involved (Figure 3B). 

**Figure 4:**
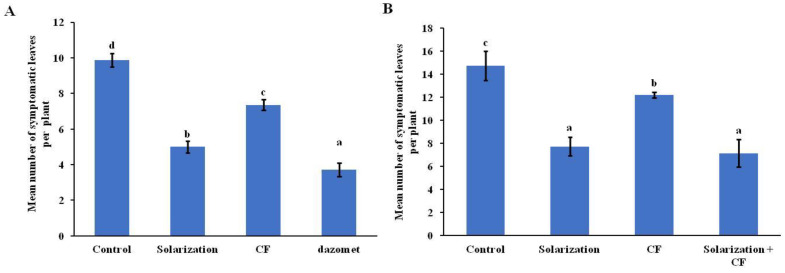
Estimation of mean number of leaves exhibiting foliar disease symptoms caused by *Fusarium equiseti* on lettuce plants grown in plots treated with several soil disinfestation methods during 2019 (**A**) and 2020 (**B**). Error bars represent the standard deviation. Means followed by different letters indicate statistically significant differences among the evaluated treatments as defined by Tukey’s test (*p* < 0.05). Control: no treatment; CF: *Coniothyrium*-based biofungicide.

As for leaf spots caused by *F. equiseti* during 2019, the highest incidence was observed in the control treatment, while the lowest was recorded in the chemical-treated plants, followed by plants collected from the solarized- and the CF-treated plots (Figure 4A). In 2020, the highest number of symptomatic leaves was observed once again in the plants collected from the control treatment (Figure 4B). On the other hand, plants collected from the solarized plots—with either the solarization applied alone or in combination with the biological control agent—showed a significantly lower number (*p* < 0.05) of leaves with symptoms compared to the control treatment (Figure 4A,B).

### 3.4. Plant Fresh Weight

**Figure 5:**
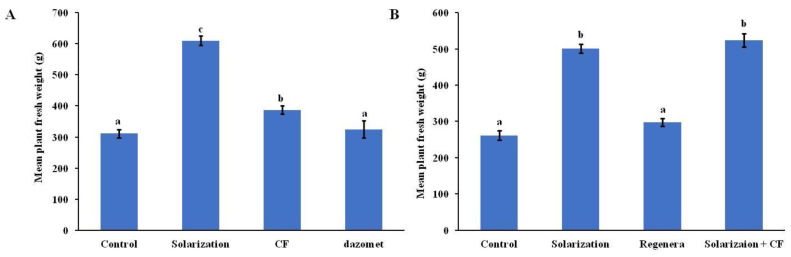
Estimation of mean fresh weight (gram) of lettuce plants grown in the plots treated with different soil disinfestation treatments during 2019 (**A**) and 2020 (**B**). Error bars represent the standard deviation. Means followed by different letters indicate statistically significant differences among the evaluated treatments as defined by Tukey’s test (*p* < 0.05). Control: no treatment; CF: *Coniothyrium*-based biofungicide.

In 2019, the highest fresh weight was recorded in plants grown in the solarized plots (Figure 5A). Lettuce plants grown in plots where the chemical treatment (dazomet) was applied did not differ significantly in their fresh weight in comparison to the untreated control (*p* > 0.05). Plants that received the CF treatment showed significantly higher fresh weight in comparison to the control and chemical-treated plants and were significantly lower than the fresh weight of plants collected from solarized plots (Figure 5A). In 2020, no significant differences were observed between the control plants and the plants collected from plots treated with the biological control agent, showing the lowest fresh weight among all treatments (Figure 5B). In contrast, the highest fresh weight was measured in the solarization-treated plants, whether this treatment was applied alone or in combination with the biological control agent (Figure 5B).

### 3.5. Microbial Community Composition and Dynamics

After quality control, an average of 449.068 high-quality sequences per sample were obtained for bacteria. The generated rarefaction curve based on the Shannon diversity index values reached a plateau in all samples, indicating that the sequencing provided adequate microbial diversity coverage (Figure S2). Filtered tags were clustered into OTUs at 97% similarity, and detailed data on the various treatments implemented are provided in Table 1.

### 3.6. α-Diversity and β-Diversity of Soil Bacterial Communities

**Figure 6:**
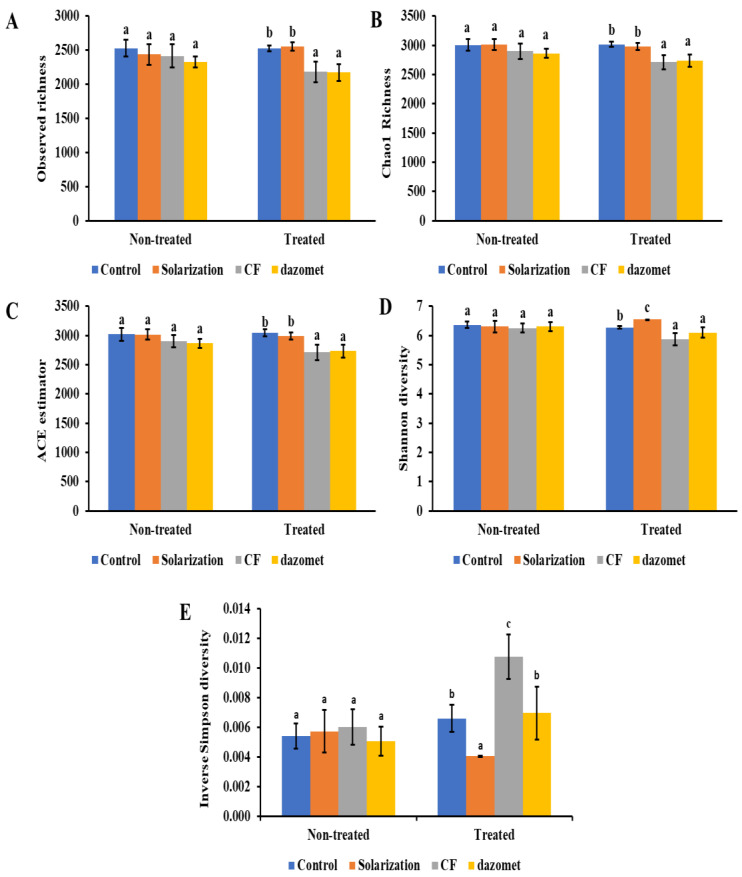
α-diversity indices of the bacterial communities estimated in this study. From top to bottom, the figure shows the OTU richness using observed (**A**), Chao1 (**B**), and ACE (**C**) indices, and diversity using the Shannon (**D**) and Inverse Simpson (**E**) indices. Means followed by different letters indicate statistically significant differences.

The analysis of variance (one-way ANOVA) of the main factor ”soil disinfestation treatment” was addressed to determine if the various treatments affected the α-diversity indices of the bacterial communities (Figure 6). Among the tested treatments, soil solarization did not induce any significant effect (*p* > 0.05) on any of the α-diversity indices measured, except for the Shannon and Simpson diversity indices, which were significantly higher and lower (*p* < 0.05), respectively, compared to the control treatment (Figure 6D,E). On the other hand, a significantly lower richness (*p* < 0.05) in terms of observed species, Chao1, and ACE indices as well as a significantly lower Shannon diversity index were observed in the disinfestation treatment with the soil fumigant dazomet in comparison to the non-treated control (Figure 6A–C). In addition, dazomet treatment did not induce any effect (*p* > 0.05) on the Inverse Simpson index (Figure 6D,E). All measured indices were significantly lower (*p* < 0.05), except for the Inverse Simpson diversity index; this was significantly higher (Figure 6E) when the treatment that included the *Coniothyrium*-based fungicide was compared with the non-treated control.

The principal component analysis (PCA) showed that the various treatments applied were the determinants of the structural composition of the bacterial communities (Figure S3). At the OTU level, soil samples collected before the application of the treatments (non-treated plots) showed a weak clustering of the bacterial communities in the first axis (Figure S3A). The first principal component (PC1) on the X-axis, and the second principal component (PC2) on the Y-axis explained 42.08 and 15.45% of the total variance, respectively. Similarity among soil samples collected from the same plot was low as the OTUs were not closely related (Figure S3A). On the other hand, PC1 explained 46.94%, and PC2 explained 22.30% of the total variance in the soil samples collected after the implementation of the treatments (treated plots). The solarization and the dazomet treatments were significantly separated from the other two conditions (control and biofungicide treatment, CF), which were highly related in the X-axis (Figure S3B). Samples from the control and the CF treatment clustered in the first quadrant, implying that the bacterial communities in these two conditions were more similar than those in the plots receiving solarization or dazomet treatments.

β-diversity analysis was additionally used to evaluate differences in individual soil samples for species complexity based on the OTUs derived from the obtained sequences. Principal coordinates analysis (PCoA) plot of Bray–Curtis dissimilarity among samples showed a significant effect of the applied treatments on the bacterial communities (Figure S4). Among samples obtained from the same treatment (treated plots), high similarity was observed and samples were clustered together. On the other hand, individual samples of the same treatment showed higher dissimilarity to the other soil treatments and to samples obtained before the application of the various treatments (non-treated plots). In addition, samples from the non-treated plots in most cases did not cluster together, showing high dissimilarity (Figure S4). These observations are in accordance with the ones deduced from the PCA analysis, which was described above.

### 3.7. Phylum- and Class-Level Taxonomic Composition Distribution

**Figure 7:**
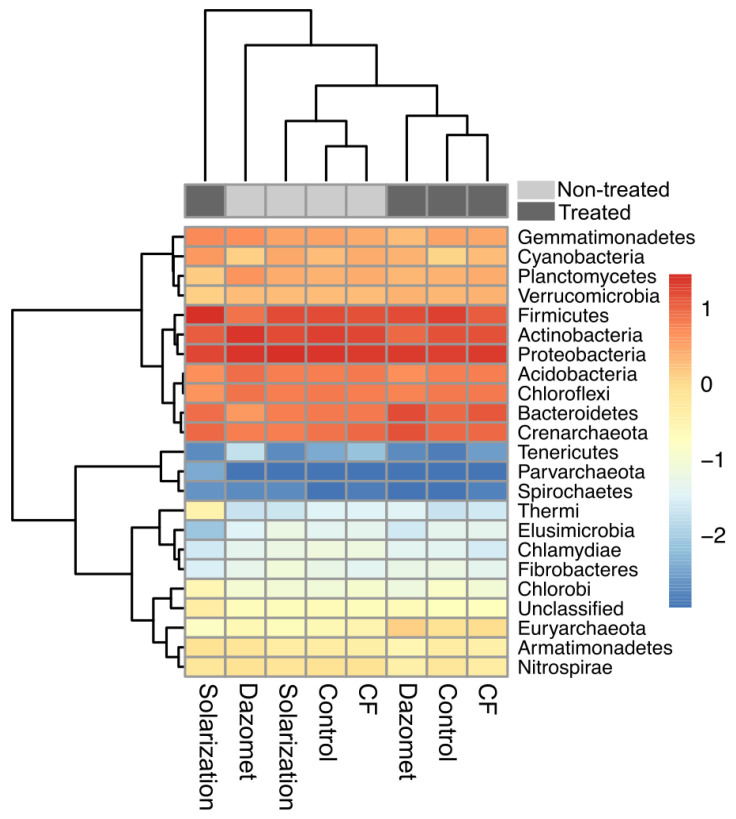
Heatmap showing the relative abundance of bacterial communities in different experimental plots, before and after the application of the different treatments (non-treated, and treated plots, respectively) on the phylum level. Rows correspond to bacterial phyla and columns represent individual treatments. Within the heatmap, each cell indicates the log_10_-transformed relative abundance of operational taxonomic units (OTUs). The color of heatmap is scaled based on relative abundance. A low abundance value is indicated by the blue color and a high value is indicated by red, as explained by the color scale at the right side of the figure.

In total, 22 bacterial phyla were identified in the soil samples (Figure 7). Across all plots, whether the soil samples were obtained from non-treated or treated plots, Proteobacteria was the dominant phylum (22.7 ± 3.27%), followed by Actinobacteria (16.5 ± 5.0%) and Firmicutes (16.1 ± 5.7%). In addition, Chloroflexi, Acidobacteria, Crenarchaeota, and Bacteroidetes were the succeeding phyla in terms of relative abundance, showing similar abundance levels, accounting for 6.5 ± 1.1, 6.5 ± 1.5, 8.9 ± 3.8, and 9.4 ± 3.5%, respectively, of the total bacterial communities (Figure 7). The relative abundance of Firmicutes increased by solarization, while the same treatment had negative effects on Proteobacteria and Actinobacteria. Interestingly, the phylum Thermi increased after the application of soil solarization. Actinobacteria, Acidobacteria, and Chloroflexi decreased by dazomet treatment, while Firmicutes and Proteobacteria increased. On the other hand, the CF treatment had no effect on the three dominant bacterial phyla and only induced an increase in the relative abundance of Bacteroidetes (Figure 7).

**Figure 8:**
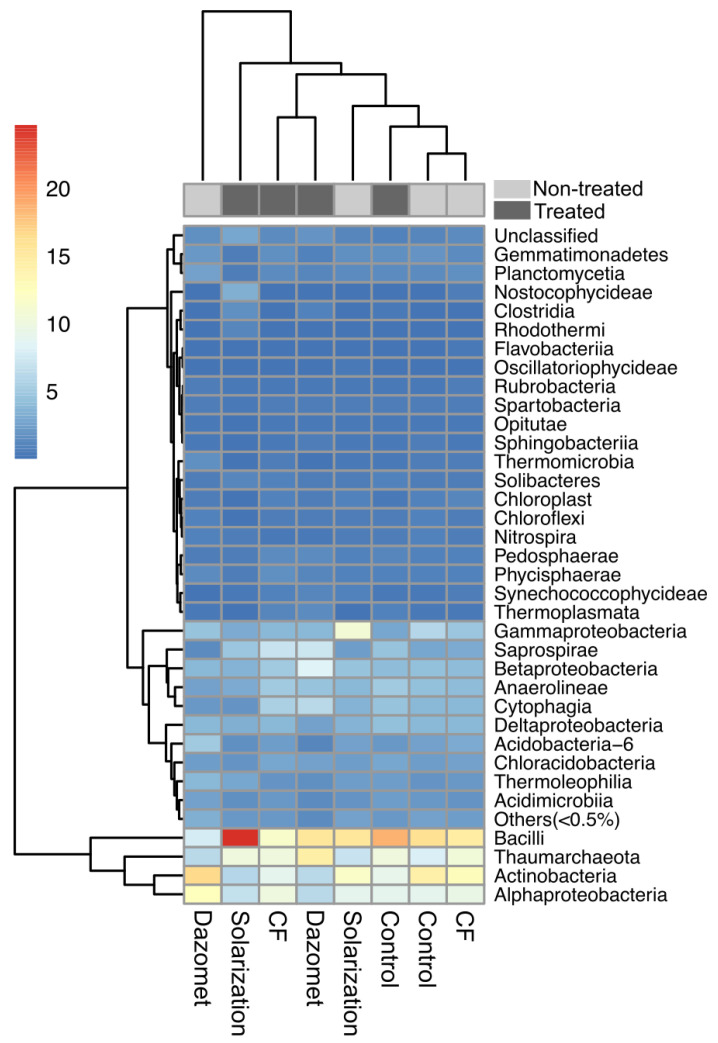
Heatmap showing the relative abundance of bacterial communities in experimental plots, before and after the application of the different soil disinfestation treatments (non-treated and treated plots, respectively) on the class level. Rows correspond to bacterial classes and columns represent individual treatments. The color of heatmap is scaled based on relative abundance. A low abundance value is indicated by the blue color and a high value is indicated by red, as explained by the color scale at the right side of the figure.

At the class level, the relative abundance of Alpha-Proteobacteria decreased in solarized and fumigated soils, while it increased in the soil that received the biofungicide application (Figure 8). In addition, the relative abundance of Beta- and Gamma-Proteobacteria increased and decreased, respectively, in all the evaluated soil treatments. The most notable observation was the effect of solarization on the relative abundance of class Bacilli, which increased from 15.5 ± 2.1% to 24.6 ± 2.2%, while the relative abundance of the same class in the plots serving as control was 16.1 ± 3.4% at the first sampling time and 18.6 ± 1.4% at the second time (Figure 8). 

### 3.8. Effect of Applied Soil Treatments on Specific Bacterial Genera 

**Figure 9:**
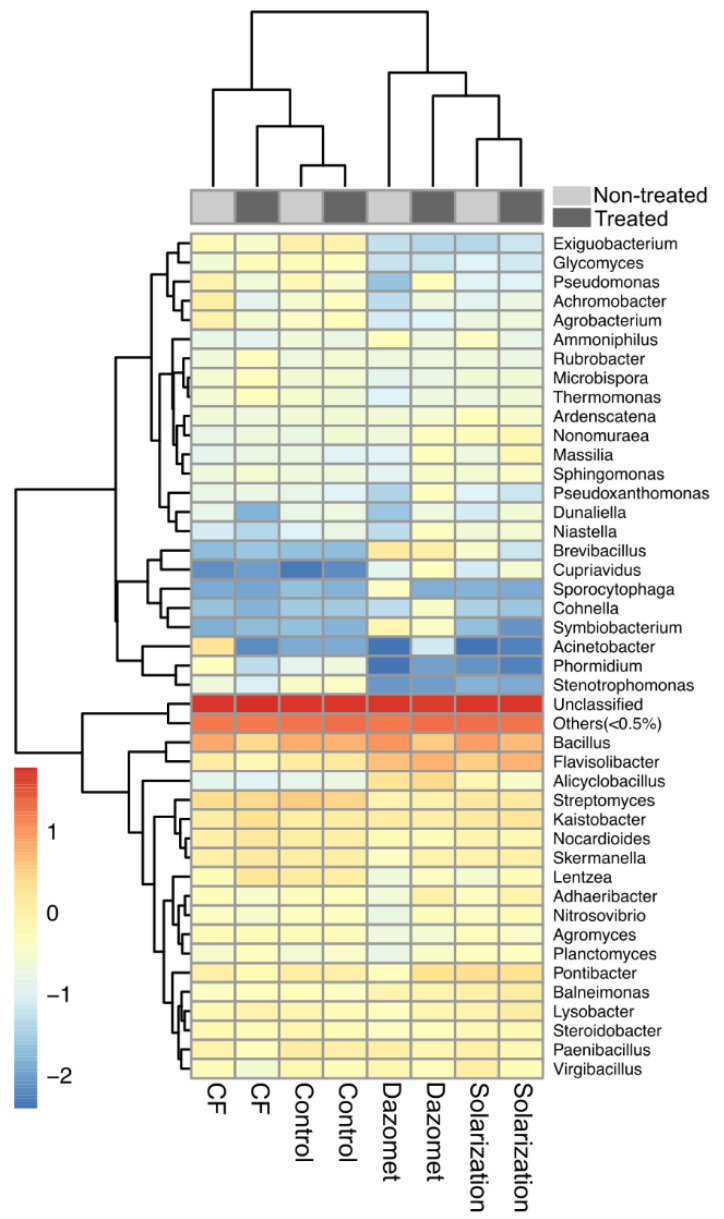
Heatmap showing the relative abundance of bacterial communities before and after the application of the different soil disinfestation treatments (non-treated and treated plots, respectively) on the genus level. Rows correspond to bacterial genera and columns represent individual treatments. Within the heatmap, each cell indicates the log_10_-transformed relative abundance of operational taxonomic units (OTUs). The color of heatmap is scaled based on relative abundance. A low abundance value is indicated by the blue color and a high value is indicated by red, as explained by the color scale at the right side of the figure.

*Bacillus* was the predominant bacterial genus in soil samples, before and after the implementation of the soil treatments (Figure 9). However, this genus was disfavored after the application of the disinfestation treatments compared to the corresponding non-treated plots. In addition, the relative abundance of genera *Brevibacillus* and *Paenibacillus*, both belonging also to the phylum Firmicutes, were also reduced in the treated plots (Figure 9). The relative abundance of the genus *Pseudomonas* (Proteobacteria) showed no difference in solarization-treated plots, while on the other hand, this genus was reduced in CF-treated plots but increased in dazomet-treated plots when compared to the corresponding non-treated plots. In the same phylum, *Massilia* values were increased in solarized and fumigated plots, while in the CF-treated ones, the values remained stable. Interestingly, the relative abundance of ammonia-oxidizing bacteria that catabolize ammonia to nitrite, such as *Nitrosovibrio* spp. (Proteobacteria), was increased in solarized and fumigated plots (especially in the latter one), while the control plots showed the same relative abundance throughout the experiment. On the other hand, *Nitrosovibrio* was disfavored in the CF-treated plots. *Glycomyces* and *Microbispora* spp., which both belong to the phylum Actinobacteria, showed a similar pattern. The relative abundance of these two genera did not change after the application of the treatments, except for the CF treatment, in which an increase was observed (Figure 9). Furthermore, no significant difference in the abundance of *Streptomyces* was observed after the application of the different soil treatments. Overall, it is important to point out that the relative abundance of all the individual bacteria genera remained unchanged in the plots serving as controls, validating the effects of the applied soil treatments on the bacterial soil communities. 

## 4. Discussion

This is the first comprehensive study aiming to identify the impact of several soil disinfestation treatments on soilborne pathogens affecting leafy vegetables such as lettuce. Quantification of the four soilborne pathogens’ inoculum with qPCR revealed that all the evaluated treatments had a significant impact on the population dynamics of the tested pathogens in comparison to the non-treated control. Among the evaluated treatments, soil solarization was the most effective, followed by the soil fumigant application and the commercial biofungicide. The limited amount of quantification data of soilborne pathogens via qPCR is due to the culture-based approach, adopted until recently, which was based on conventional plating of soil suspensions on selective or semi-selective media along with observation of morphological characteristics [34]. However, our results are in agreement with the results of previous studies where *R. solani* and *F. oxysporum* populations in the soil decreased more than 90% after 6-week solarization in strawberry or tomato cultivations [22,35,36]. The evaluation of the effect of solarization on several soilborne pathogens, including *F. oxysporum* and *P. ultimum*, in a spinach cropping system using qPCR showed a significant decrease in their abundance after the application of the specific treatment [37].

In both years of the experiment, the *Coniothyrium*-based biofungicide induced a significantly lower decrease in the populations of the four evaluated soilborne pathogens compared to the solarized plots. However, when this biofungicide was applied in combination with solarization, the results showed lower pathogen populations compared with the solarization-treated plots, implying a potential synergistic mode of action between these two treatments. So far, another *Coniothyrium* species named *C. minitans* has been developed as a commercial biological control agent for the control of fungal diseases caused by *Sclerotinia sclerotiorum* and *S. minor*, such as Sclerotinia lettuce drop, in greenhouses and in the field [38]. Commercial formulation of *C. minitans* has already been observed to efficiently control Sclerotinia disease in several high-value crops [39]. Combined application of *C. minitans* with heat-treated soil or a low dose of several fungicides resulted in a lower number of viable sclerotia, and significant reduction in Sclerotinia disease symptoms on beans, respectively, compared to the results observed when its control measure was applied individually, implying the potential use of this commercial product in an integrated pest management system [40,41]. 

The suppressive effect of dazomet application on various soilborne pathogens has already been observed in previous studies [42,43,44,45]. In our study, the chemical fumigant dazomet significantly reduced the population of the four soilborne pathogens in comparison to the non-treated plots, especially in the case of *R. solani*, which was reduced in similar levels to those observed in the case of solarization. Although, *P. ultimum* was also significantly reduced compared with the non-treated plots, solarization was observed to be the most effective treatment against the specific soilborne pathogen. Solarization and dazomet also significantly reduced the populations of the two *Fusarium* spp. compared with those observed in the non-treated plots, however, to lower levels compared to *R. solani* and *P. ultimum*. Both *Fusarium* species have the ability to produce resting structures (chlamydospores), which are considered resistant to solarization [46] and chemical fumigation treatments, especially when they are formed on crop debris [47]. Nonetheless, at the harvest stage, dazomet showed the lowest significant disease incidence in the case of estimation of *F. equiseti* disease symptoms, followed by solarization. In line with this observation, Bennett [46] reported that chlamydospores that have survived heat treatment present reduced virulence, implying that solarization might be more effective in reducing disease incidence than the estimation of the *Fusarium* population indicates, which might also explain the impact of dazomet on the recorded disease incidence.

Problems, such as phytotoxicity after the application of dazomet, have been recorded for a long time [48]. Ren et al.’s study [49] provides support that uneven distribution results in higher concentrations in the soil, which are phytotoxic to plants. In addition, phytotoxic effects on plants are also observed when dazomet is applied in large particle sizes that prolong its half-life, exhibiting a lower degradation rate and thus leading to crop phytotoxicity [49,50]. Interestingly, such an effect was observed after the application of dazomet in the first year of the experiment carried out in this study. Lettuce plants grown in dazomet-treated plots showed limited plant growth, and their fresh weight was similar to the one estimated for plants grown in non-treated plots. Most likely, this impact is attributed to dazomet residues remaining in the soil, and the subsequent almost-immediate cultivation of lettuce plants after removal of the plastic film. Dazomet residues remain in the soil more than a month after its application, even under suitable soil conditions and using the adequate application dose [49]. Hence, a longer soil aeration period after fumigation is crucial before cultivation [50].

In the second year of the experiment, the findings from the qPCR assays were supported by the disease assessment, which was carried out for two soilborne pathogens (*R. solani* and *F. equiseti*). More specifically, lettuce plants grown in solarized plots showed the lowest damping-off caused by *R. solani*, incidence (%), and the lowest number of leaves exhibiting foliar disease symptoms caused by *F. equiseti*, compared to the remaining disinfestation treatments. In accordance with the population estimation via qPCR, the efficacy of solarization appeared to be improved when combined with biofungicide. However, in the first year of the experiment, the lowest disease assessment results were recorded from plants grown in dazomet-treated plots, followed by the results obtained from plants cultivated in plots that received the application of solarization and biofungicide. Most likely, the residual action of dazomet, which has been observed to remain in soil for a prolonged period [49], might have resulted in higher control effectiveness against these two soilborne diseases, causing at the same time phytotoxicity to the lettuce plants as mentioned above. The phytotoxic effect recorded after the application of dazomet, the notable results of solarization, and the enhanced results from the combination of solarization and biofungicide denote the implementation of alternative soil disinfestation methods in the frame of an integrated pest management system [18]. 

In addition to the highest reduction in pathogen populations and the higher disease control efficacy observed in solarized soils, in these plots, the highest fresh weight for the produced lettuce plants was also observed. The increased fresh weight of the plants suggests that solarization positively affected their growth. In general, soil solarization is considered to increase the yields and quality of several vegetable crops due to a direct effect on the control of many soilborne pathogens [51]. For instance, soil solarization effectively suppressed Fusarium wilt in lettuce systems, increasing, at the same time, head weight and total yield [52]. Increased growth responses in lettuce production systems were also reported in other studies in which solarization was applied [53,54,55]; however, these studies showed that in addition to the direct effect on the growth because of the high disease control efficacy, the increased plant growth was also associated with an alteration in soil microbial populations that favored beneficial microorganisms such as bacteria of the genera *Pseudomonas* and *Bacillus*.

Subsequently, in our study, the effect of the evaluated soil disinfestation methods on soil bacterial community structure was investigated. The results showed that the three tested methods had an impact on the soil bacterial microbiome structure, affecting the relative abundance of the predominant bacterial phyla. Solarization increased the relative abundance of Firmicutes and had negative effects on the relative abundance of Proteobacteria and Actinobacteria. On the other hand, Firmicutes and Proteobacteria increased, and Actinobacteria decreased in soil treated with the chemical fumigant dazomet. In contrast, soil treatment with biofungicide had no significant effects on the predominant bacterial phyla, which is not surprising as it is considered a fungus-specific biofungicide [56]. Similar effects of solarization on Firmicutes and Actinobacteria have also been recorded by Kanaan et al. [57], while Hestmark et al. [58] noticed an increase in relative abundance of phylum Firmicutes and a decrease in Proteobacteria. Moreover, total bacteria and α-Proteobacteria were significantly reduced in solarized experimental plots in fields cultivated with strawberry, while *β-Proteobacteria, Firmicutes*, and *Actinobacteria* were significantly increased [35]. Contrasting results related to the effect of dazomet applications in soil on the composition of the bacterial community have been recorded in the review study of Castellano-Hinojosa et al. [59], who refer that the treatment with dazomet has been shown to either not affect, increase, or reduce the abundance and diversity of soil bacterial communities after fumigation compared to non-treated soils. These inconsistencies in the effects of dazomet may be attributed to differences in the applied dose, soil physicochemical properties, and/or environmental conditions that affect the degradation rates of the specific soil fumigant [59].

The higher lettuce productivity recorded in the solarized plots may be explained by the changes in the bacterial community described above, which could have affected plant growth by means of changes in nutrient availability, suppression of soilborne pathogens, and/or increase in the relative abundance of plant-growth-promoting rhizobacteria [60]. The high temperatures prevailing during solarization may lead to an increase in thermophilic or thermotolerant bacteria [61]. The higher relative abundance of Firmicutes, especially after the application of solarization, may be explained by their spore-mediated thermotolerance, while the lower abundance of Acidobacteria and Proteobacteria might be associated with the observed relative thermosensitivity in these groups [57].

Although in this study, the α-diversity of bacteria showed similar richness after solarization with that observed in non-solarized plots, the taxonomic composition of bacterial communities has been modified after the application of solarization. Such a finding is in accordance with those reported in previous studies [57,61]. Interestingly, in the present study, the relative abundance of class Bacilli was remarkably increased after solarization. Class Bacilli consists mainly of thermotolerant bacteria, and the temperatures developed during solarization usually have a beneficial impact on their populations and, at the same time, play a significant role in the disease suppressiveness observed in solarized soils [4].

The relative abundance of *Streptomyces* increased in all treated plots, although not significantly compared to the non-treated ones. However, *Streptomyces* is a well-known growth-promoting bacterium in the rhizosphere of plants [62], also being responsible for the production of antibiotics for biological control [63,64]. Moreover, rhizobacteria, such as *Bacillus* and *Pseudomonas* spp., exhibit antagonistic effects and trigger systemic resistance in plants [65]. *Bacillus*, although negatively affected in all the treated plots, is also known as a genus that includes beneficial species that improve soil nutrition and suppress plant diseases [66]. On the other hand, the genus *Pseudomonas*—which was not affected in solarized plots, increased after fumigation, and decreased in the CF-treated plots—includes bacteria species that promote plant growth and suppress soilborne diseases [67].

## 5. Conclusions

The results of our study indicate that the application of solarization as a soil disinfestation method induced the most significant decrease in the population of the four most important soilborne pathogens affecting lettuce. The reduction in the pathogen inoculum in the treated plots was directly correlated with a strong reduction in the incidence of diseases caused by *R. solani* and *F. equiseti*, while, in addition, solarization increased the fresh weight of lettuce plants at harvesting. It can also be concluded that the applied treatments had an impact on the richness and diversity of the soil bacterial communities. Further studies are needed to evaluate the short- and long-term shifts in microbial communities, especially after the cultivation of crop plants in treated soils. Such studies could provide a better understanding of the factors that affect disease control and shape the bacterial community structure and lead to the implementation of the most suitable soil disinfestation measure or measures. The pre-planting inoculum density, as well as soil health, determine the effectiveness of the implemented practices, especially in intensive cropping systems such as those of leafy vegetables. The challenging task of maintaining healthy soil is to significantly reduce soilborne pathogens and sustain or even enhance potential beneficial microorganisms from the indigenous microbiome after certain management schemes. Overall, the understanding of the effects of soil disinfestation methods on the suppression of given soilborne pathogens could form the basis for the adoption of the most effective management strategy. 

## Figures and Tables

**Table 1:** Number of operational taxonomic units (OTUs) per treatment at the two sampling times; before and after implementation of the various soil disinfestation treatments.

Treatment	Plots	
	Non-Treated	Treated
Control	2530 ± 154.73	2418 ± 206.44
Solarization	2526 ± 56.96	2126 ± 188.25
CF	2437 ± 187.35	2329 ± 100.78
Dazomet	2617 ± 77.02	2094 ± 102.53

## Data Availability

The data presented in this study are available in the article.

## Supplementary Materials

The following supporting information can be downloaded at: https://www.mdpi.com/article/10.3390/biology13080624/s1, Figure S1: Standard curve for (A) *Rhizoctonia solani*, (B) *Pythium ultimum*, and (C) *Fusarium oxysporum* in a RT-qPCR assay. Cycle threshold (Ct) values were plotted against the plasmid DNA serial-dilution concentrations prepared with sterilized water. Each dot represents the mean value of three replicates. Error bars represent the standard deviation. Most error bars were too small to be illustrated; Figure S2: Rarefaction curve for the Shannon index of soil samples collected from the experimental field plots and analyzed for their bacterial microbiome; Figure S3: Principal component analysis (PCA) based on the OTU levels for soil samples collected from (A) non-treated (Group 1–4) and (B) treated (Group 5–8) plots. Group 1,5 = control; Group 2,6 = solarization; Group 3,7 = CF (*Coniothyrium*-based biofubgicide); and Group 4,8 = dazomet. X-axis represents the 1st principal component (PC1), and Y-axis represents the 2nd principal component (PC2). Numbers in brackets represent contributions of principal components to differences among samples. A dot represents each sample, and different colors represent different groups; Figure S4: Principal coordinates analysis (PCoA) plot of Bray–Curtis dissimilarity among soil samples obtained before (non-treated) and after the application of the various soil disinfestation treatments (treated). Blue color represents exact similarity in community structure and red color indicated the highest dissimilarity among samples, as explained by the color scale at the right side of the figure.
