# Supplementary material for: Soil Solarization Efficiently Reduces Fungal Soilborne Pathogen Populations, Promotes Lettuce Plant Growth, and Affects the Soil Bacterial Community
Source: Biology (Basel). 2024 Aug 15;13(8):624. doi: 10.3390/biology13080624 (PMC11352132; doi:10.3390/biology13080624)
Supplement: Supplementary file 1 [file biology-13-00624-s001.zip › biology-3145933-supplementary.pdf]

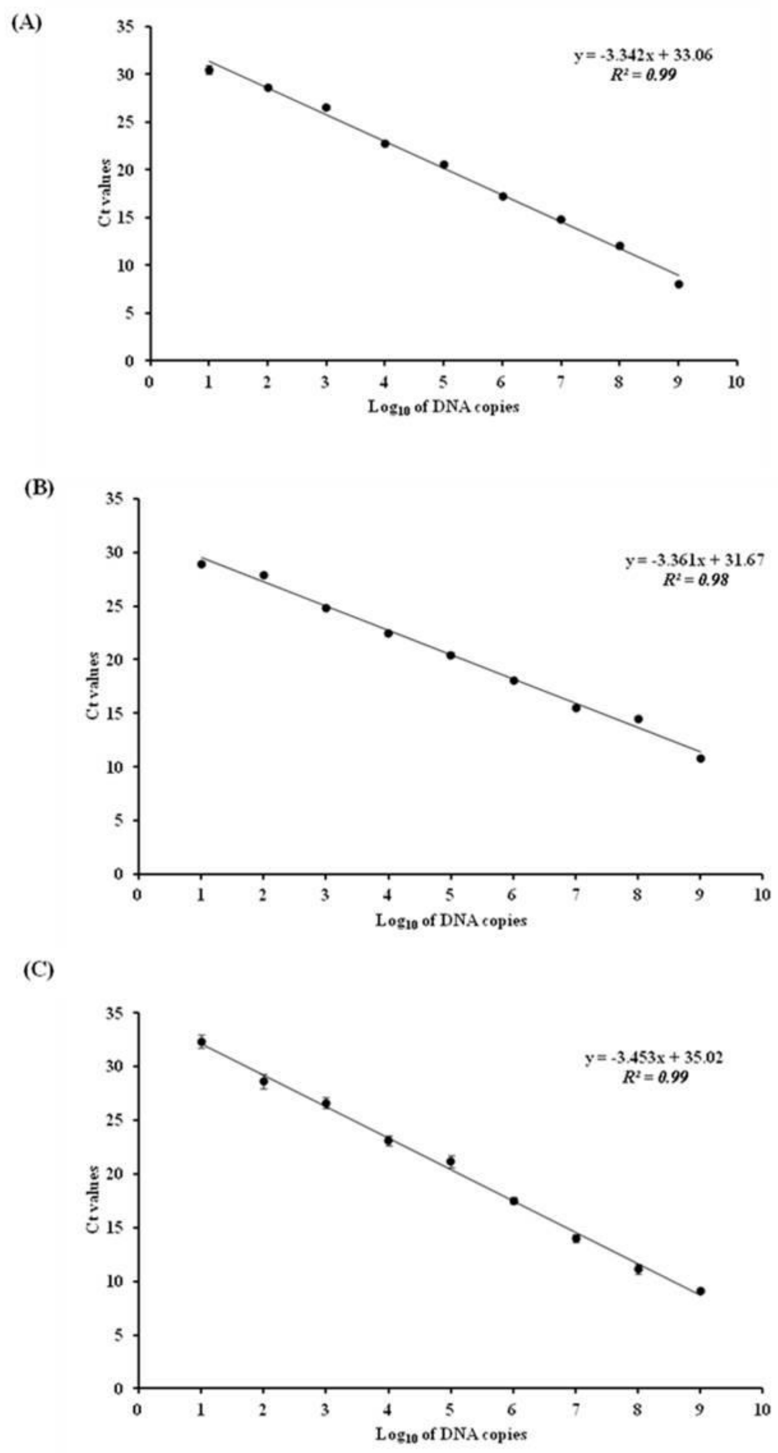

Figure S1. Standard curve for (A) *Rhizoctonia solani*, (B) *Pythium ultimum*, and (C) *Fusarium oxysporum* in a RT-qPCR assay. Cycle threshold (Ct) values were plotted against the plasmid DNA serial-dilution concentrations prepared with sterilized water. Each dot represents the mean value of three replicates. Error bars represent the standard deviation. Most error bars were too small to be illustrated.

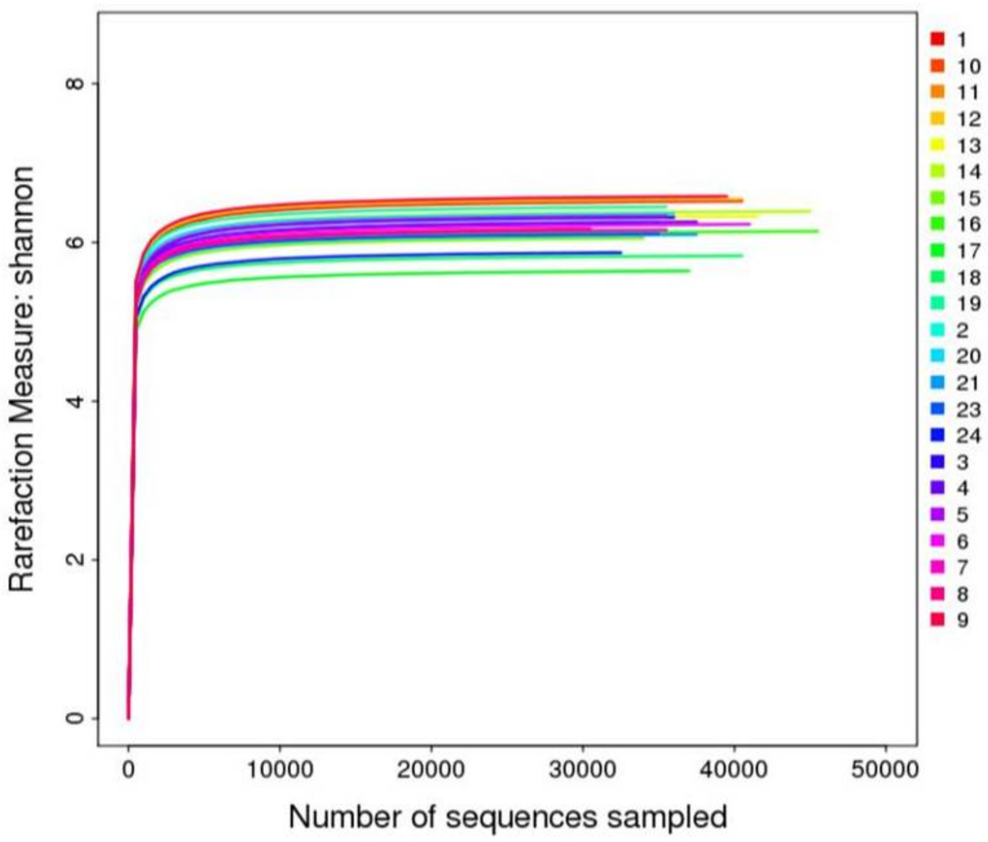

Figure S2. Rarefaction curve for Sannon index of soil samples collected from the experimental field' plots and analyzed for their bacterial microbiome.

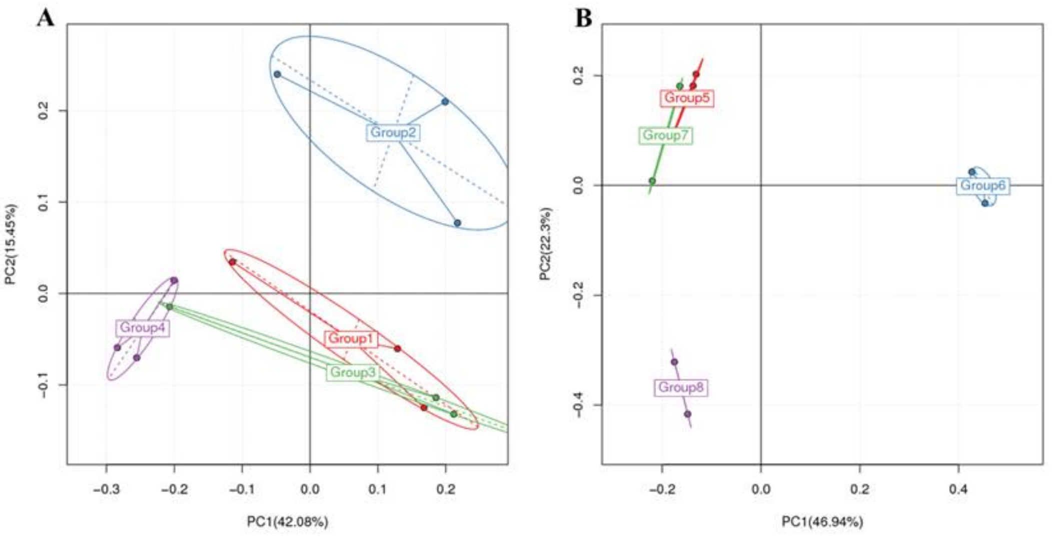

Figure S3. Principal component analysis (PCA) based on the OTU levels for soil samples collected from (**A**) non-treated (Group 1-4), and (**B**) treated (Group 5-8) plots. Group 1,5 = Control; Group 2,6 = solarization; Group 3,7 = CF (*Coniothyrium*-based biofubgicide); and Group 4,8 = dazomet. X-axis represents the 1st principal component (PC1), and Y-axis represents the 2nd principal component (PC2). Numbers in brackets represent contributions of principal components to differences among samples. A dot represents each sample, and different colors represent different groups.

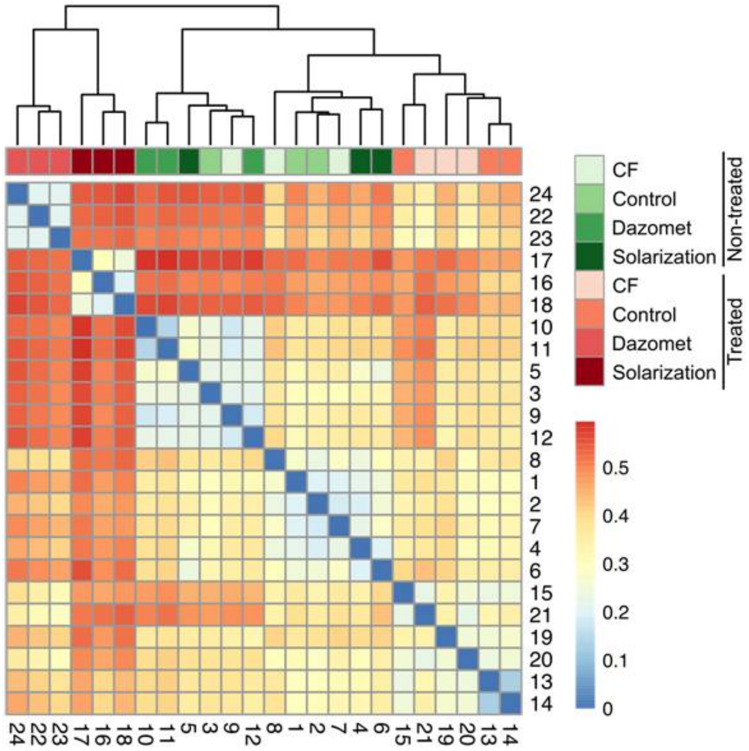

Figure S4. Principal coordinates analysis (PCoA) plot of Bray–Curtis dissimilarity among soil samples obtained before (Non-treated) and after the application of the various soil disinfestation treatments (Treated). Blue color represents exact similar community structure and red color indicated the highest dissimilarity among samples, as explained by the color scale at the right side of the Figure.
